# Antiarrhythmic Mechanisms of Chinese Herbal Medicine Dingji Fumai Decoction

**DOI:** 10.1155/2020/9185707

**Published:** 2020-03-20

**Authors:** Bo Liang, Yan Zhou, Ling Fu, Hui-Ling Liao

**Affiliations:** ^1^Nanjing University of Chinese Medicine, Nanjing, China; ^2^Zigong Hospital of TCM, Zigong, China; ^3^Chongqing Traditional Chinese Medicine Hospital, Chongqing, China; ^4^College of Integrated Traditional Chinese and Western Medicine Hospital (T.C.M.) Affiliated to Southwest Medical University, Luzhou, China

## Abstract

**Background:**

Dingji Fumai decoction (DFD) is used to treat ventricular arrhythmia, and it has provided a very good curative effect. However, its cellular electrophysiological mechanism is unknown.

**Methods:**

Electrocardiogram was recorded, and oxidative stress response and ion-channel-related molecules were detected in rats with barium chloride- and aconitine-induced ventricular arrhythmia. Moreover, whole-cell patch-clamp assay was used to investigate the inhibitory effect of DFD on Nav_1.5_ in Chinese hamster ovary cells.

**Results:**

DFD prolonged the occurrence time and shortened the duration of ventricular arrhythmia, decreased the malondialdehyde and increased the superoxide dismutase, and alleviated the activation of Na^+^-K^+^-ATPase and connexin-43. DFD suppressed Nav_1.5_dose-dependently with an IC_50_ of 24.0 ± 2.4 mg/mL.

**Conclusions:**

The clinical antiarrhythmic mechanisms of DFD are based on its antioxidant potential, alleviation of Na^+^-K^+^-ATPase and connexin-43, and class I antiarrhythmic properties by suppressing Nav_1.5_dose-dependently with an IC_50_ of 24.0 ± 2.4 mg/mL.

## 1. Introduction

Cardiac arrhythmia is a group of conditions in which the heart beats irregularly [1]. Although some arrhythmias are not serious because many arrhythmic patients have no symptoms, some patients are prone to developing fatal or nonfatal complications, including heart failure, stroke, and cardiac arrest.

A large number of ongoing studies have attempted to gain a better understanding of cardiac arrhythmia [2] and have found solutions to cure it. Pharmaceutical agents that work well and have fewer side effects are also being explored; however, the progress is far from satisfactory [3, 4]. Drug development for ion channels has been relatively mature for nearly half a century, but the results of new drugs are not very encouraging because of the adverse reactions [5].

Dingji Fumai decoction (DFD) is an empirical prescription developed by Professor Luo, a national tutor, according to the basic theory of traditional Chinese medicine for the treatment of palpitation (also known as arrhythmia in modern medicine). DFD, consisting of *Chuanxiong Rhizoma* (Chuanxiong), *Jujubae Fructus* (Dazao), *Poria cocos* (Schw.) *Wolf* (Fuling), *Cinnamomi Ramulus* (Guizhi), *Silktree Albizia Bark* (Hehuanpi), *Osdraconis (Fossiliaossiamastodi)* (Longgu), *Ostrea Gigas Thunberg* (Muli), *Ziziphi Spinosae Semen* (Suanzaoren), *Radix Polygalae* (Yuanzhi), and *Licorice* (Gancao), is a multiherbal medicine for ventricular arrhythmia (VA), especially ventricular premature contraction (VPC) [6]. Its co-components with Xinsuning are *Poria Cocos* (Schw.) *Wolf* and *Licorice*, which significantly restrain arrhythmias induced by the chemical reagents [7]. Our previous research showed that DFD combined with metoprolol is more effective than metoprolol alone in the treatment of VPC, and there are no side effects [6]. Here, we report the effect of DFD on the electrocardiogram (ECG) of rats with barium chloride- and aconitine-induced VA, antioxygen free radical, Na^+^-K^+^-ATPase and connexin-43 (Cx43), and Nav_1.5_ sodium channel to elicit the antiarrhythmic mechanisms of DFD to provide counseling for clinicians and clinical researchers. We also hope to make a contribution to the mechanisms study of the clinically safe and effective multicomponent Chinese herbal medicine.

## 2. Methods

### 2.1. Reagents and Animals

Chinese herbs that constitute DFD were provided by the Traditional Chinese Medicine Pharmacy, Hospital (T.C.M.) Affiliated to Southwest Medical University (Luzhou, China) and were authenticated by two deputy director pharmacists. Metoprolol was purchased from AstraZeneca (London, England). Aconitine and barium chloride were purchased from Must Biotechnology (Chengdu, China) and Thermo Fisher Scientific (Heysham, England), respectively. All other chemicals were purchased from Sigma-Aldrich (St. Louis, MO, USA). Sprague-Dawley rats (180 g–220 g), obtained from the Animal Experiments Center of Southwest Medical University, were housed under standard conditions. All 40 rats were randomly divided into the following 4 groups: DFD group treated by gavage of 17.6 g/kg DFD [8], metoprolol group treated by gavage of 5.2 mg/kg metoprolol, and blank group and control group treated by gavage of 10 mg/kg normal saline once a day for 14 days. At the end of the experiment, all rats were killed by neck fracture. All experiments were reviewed and approved by the Ethics Committee on Animal Experiments and were carried out under the Guidelines for Animal Experiments at the Animal Experiments Center of Southwest Medical University (SCXK2013-17).

### 2.2. ECG Recording

The animal model was established based on previous studies [2, 9]. In brief, rats were anesthetized with 1% pentobarbital 30 mg/kg intraperitoneally, and the anesthetized rats were fixed on the plank in a prone position. Acupuncture needles were inserted subcutaneously into the medial part of limbs of rats to connect the electrodes and BL-420F Biological function experiment system (Taimeng, Chengdu, China). The normal II lead ECG was recorded, and the baseline was adjusted to zero. After the ECG was stable, rats in the other three groups, except those in the blank group, were immediately injected with either 0.4% barium chloride 4 mg/kg or 0. 001% aconitine 30 ug/kg through the caudal vein, and the same volume of 0.9% normal saline was injected into rats in the blank group. Finally, ECGs were recorded for 20 minutes. The following parameters were measured: types, occurrence, and duration of various VAs.

### 2.3. Detection of Molecules

Abdominal aorta blood samples were taken and freeze-thawed; and heart tissues were mixed with 10-fold volume PBS, freeze-thawed thrice, and crushed by a homogenizer. Then all specimens were centrifuged at 10 000 g for 8 min, and detection of malondialdehyde (MDA, one of the most important products of membrane lipid peroxidation) and superoxide dismutase (SOD, the natural enemy of oxygen free radicals) in the supernatant of blood samples and Na^+^-K^+^-ATPase in the supernatant of heart tissues was performed using the corresponding assay kit (Jiancheng, Nanjing, China).

### 2.4. Immunohistochemistry Assays

Before immersion in 0.05 mol/L PBS containing 30% sucrose for cryoprotection, heart tissues were fixed with 4% paraformaldehyde for 24 h. Then, the frozen tissues were cut into 25 μm sections for immunohistochemical staining using a Cx43 polyclonal antibody (Abcam, Cambridge, UK). Positive cells were observed with an Olympus microscope (Tokyo, Japan), and six fields were randomly selected to record the number of positive cells in each group.

### 2.5. Cell Culture

The Chinese hamster ovary (CHO) cell line stably expressing human cardiac Nav_1.5_ channel (Haiwei, Qingdao, China) was grown in F-12 Nutrient Mixture (Gibco, California, USA) containing 10% fetal bovine serum (FBS, Gibco) and 600 *μ*g/mL G-418 (Gibco) at 37°C in 5% CO_2_-enriched air. In each experiment, a coverslip was removed and cleaned twice with bath solution, and then it was placed in an inverted microscope stage. Nav_1.5_ current was recorded by the whole-cell patch-clamp technique.

### 2.6. Solutions and Materials

The standard bath solution contained the following (in mmol/L) : NaCl 137, KCl 4, CaCl_2_ 1.8, MgCl_2_ 1, glucose 10, and HEPES 10, while the standard pipette solution contained the following (in mmol/L) : KF 100, KCl 40, MgCl_2_ 2, EGTA 5, and HEPES 10. The pH was adjusted to 7.4 with NaOH and 7.2 with KOH, respectively, and the osmolality was all adjusted to 300 mOsm with sucrose.

Chinese herbs that constituted DFD were soaked in an eightfold volume distilled water at room temperature (20°C–25°C) for 1 h and filtered for 30 min, and then sixfold volume distilled water was added to the filter residue for 30 min filtration. The filtrate obtained after two sessions of decoction before and after merging was concentrated under vacuum, filtered by 0.22 *μ*m filter, and used as a stock solution of 2.4^*∗*^10^3^ mg/mL, which was frozen and stored at –80°C after sterilization. Then, the herbs were diluted with bath solution to obtain the desired concentrations.

Lidocaine (Sigma-Aldrich) was dissolved with dimethyl sulfoxide to obtain 100 mmol/L stock solution, which was stored at –20°C, and then it was diluted with the extracellular solution to achieve 0.1 mmol/L as the positive control. At the final dilution, dimethyl sulfoxide concentration was less than 0.1%, which had no effect on Na^+^ current [10].

### 2.7. Patch-Clamp Recording

The experiments were conducted at room temperature and performed as described previously with some modifications [10]. The borosilicate glass microelectrode tip had a resistance of 1–2 MΩ. After whole-cell recordings were achieved, the capacitance and resistance were compensated. The current signal was filtered at 8 kHz and sampled at 10 kHz. After perfusing the cells with bath solution for about 10 min to stabilize Na^+^ current, the bath solution was transferred to either the control or the compound-containing solution.

### 2.8. Statistical Analysis

Differences between two groups were analyzed by Student's two-tailed *t*-test, and multiple groups were analyzed by one-way analysis of variance (SPSS, Inc., Chicago, IL, USA). *P* values less than 0.05 were considered statistically significant.

## 3. Results

### 3.1. DFD Prolonged the Occurrence Time and Shortened the Duration of VA

**Figure 1:**
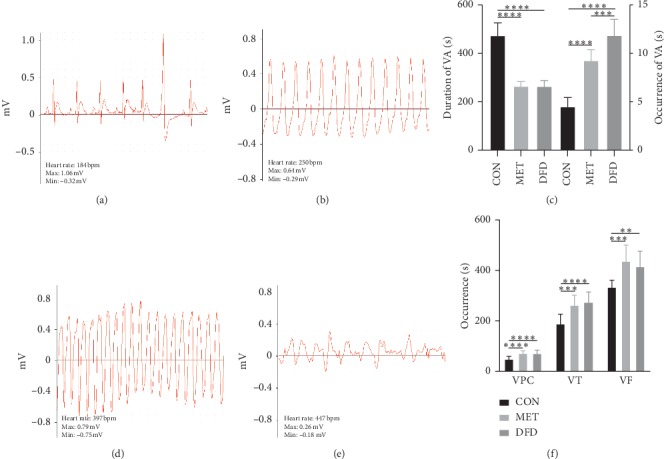
Effect of DFD on ECG in rats with barium chloride- and aconitine-induced VA. (a) An image of barium chloride-induced VPC. (b) An image of barium chloride-induced VT. (c) Occurrence and duration of VA in rats with barium chloride-induced VA. (d) An image of aconitine-induced VT. (e) An image of aconitine-induced VF. (f) Occurrence time of VPC, VT, and VF. ^*∗∗*^*P* < 0.01,^*∗∗∗*^*P* < 0.001,^*∗∗∗∗*^*P* < 0.0001.

Firstly, experiments were carried out to test the eﬀect of DFD on ECG after dose conversion from human to animal studies [8]. There were no arrhythmias in the blank group. In rats with barium chloride-induced VA, VPC occurred in all cases (Figures 1(a) and 1(b)). Compared with the control group, DFD and metoprolol prolonged the occurrence and shortened the duration of VA. The time did not show any signiﬁcant difference between the DFD group and the metoprolol group (Figure 1(c)).

Besides, in rats with aconitine-induced VA, VPC, ventricular tachycardia (VT), and ventricular fibrillation (VF) occurred successively (Figures 1(d) and 1(e)). Compared with the control group, in the same manner, DFD and metoprolol prolonged the occurrence of arrhythmia. The time did not show any signiﬁcant alteration between the DFD group and the metoprolol group (Figure 1(f)).

### 3.2. DFD Had the Effect of Antioxygen Free Radicals

**Figure 2:**
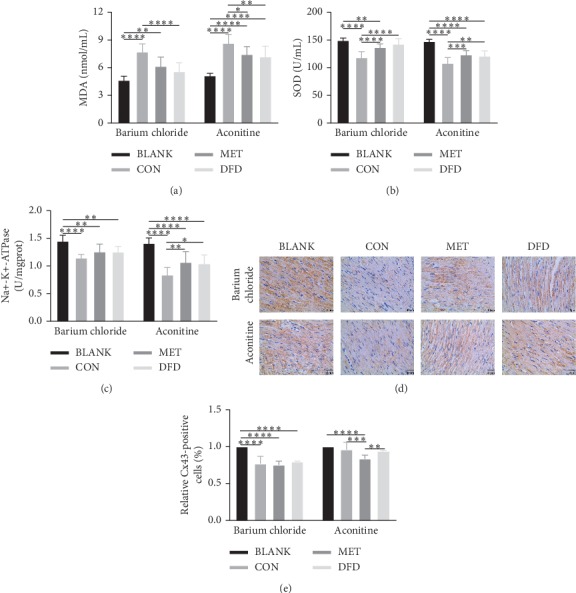
DFD exerts the effect of antioxygen free radicals via electrophysiological protection. (a) Effect of DFD on MDA in rats with barium chloride- and aconitine-induced VA. (b) Effect of DFD on SOD in rats with barium chloride- and aconitine-induced VA. (c) Effect of DFD on Na^+^-K^+^-ATPase in rats with barium chloride- and aconitine-induced VA. (d) Immunohistochemistry of Cx43 in the heart tissues; black bar represents 40 μm. (e) Quantitative analysis of Cx43-positive cells in the heart tissues. ^*∗*^*P* < 0.05,^*∗∗*^*P* < 0.01,^*∗∗∗*^*P* < 0.001,^*∗∗∗∗*^*P* < 0.0001.

The expression levels of oxidative stress response-related molecules, MDA and SOD, in the serum of rats with barium chloride-induced VA were changed 1.7-fold and 0.8-fold, respectively, compared with those in the blank group. The administration of DFD and metoprolol alleviated these changes (Figures 2(a) and 2(b)). Similar results were obtained in rats with aconitine-induced VA (Figures 2(a) and (b)).

### 3.3. DFD May Have an Effect on Ion Channels

The activity of Na^+^-K^+^-ATPase was detected. The activity of Na^+^-K^+^-ATPase was decreased 0.8-fold after barium chloride injection, and DFD and metoprolol treatment alleviated the decrease (Figure 2(c)). In addition, the results after aconitine injection were the same (Figure 2(c)). Cx43 was detected by immunohistochemical staining, and histological results were obtained by the pathologists from Southwest Medical University. The quantity of Cx43-positive cells was reduced in the barium chloride control group, the metoprolol group, and the DFD group by 23%, 20%, and 25%, respectively (Figures 2(d) and 2(e)). Moreover, the number of activated Cx43 was decreased by 1% after aconitine injection, and DFD and metoprolol alleviated the activation of Cx43-positive cardiac myocytes by 93% and 83%, respectively (Figures 2(d) and 2(e)).

### 3.4. DFD Inhibited the Sodium Current Dose-dependently

**Figure 3:**
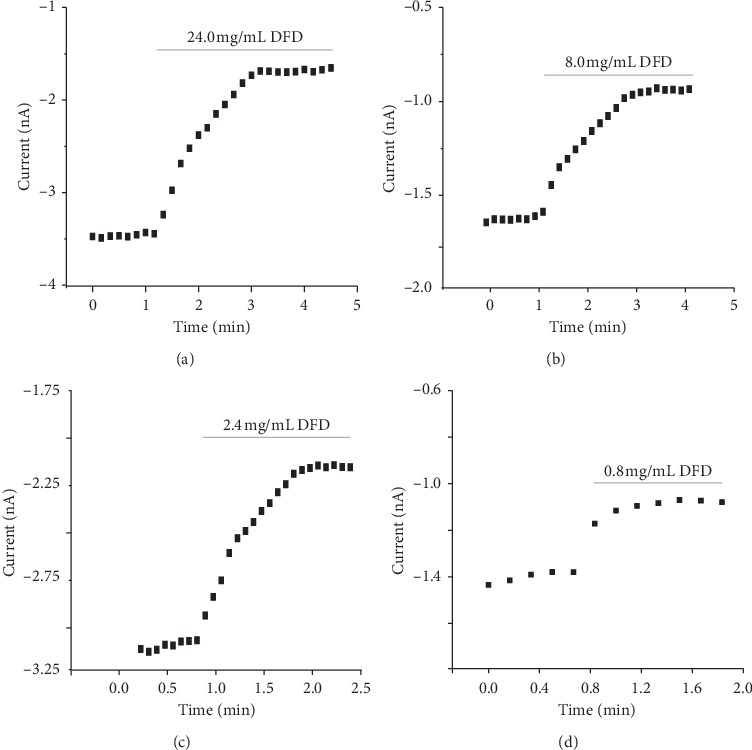
Time-history diagram of the effect of different DFD concentrations on Nav_1.5_ current in CHO cells. (a) Time-history diagram of the effect of 24.0 mg/mL DFD on Nav_1.5_ current in CHO cells. (b) Time-history diagram of the effect of 8.0 mg/mL DFD on Nav_1.5_ current in CHO cells. (c) Time-history diagram of the effect of 2.4 mg/mL DFD on Nav_1.5_ current in CHO cells. (d) Time-history diagram of the effect of 0.8 mg/mL DFD on Nav_1.5_ current in CHO cells.

**Figure 4:**
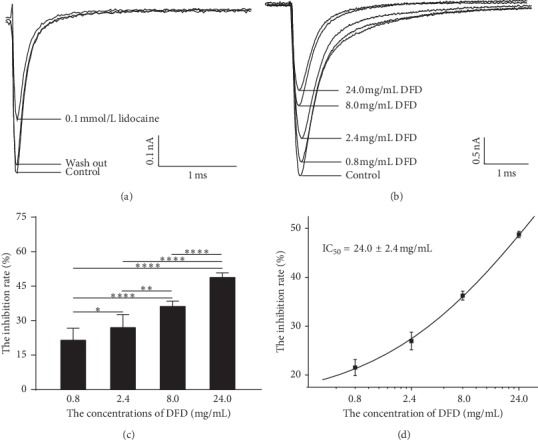
Inhibitory effect and the dose-effect curve of different DFD concentrations on Nav_1.5_ current in CHO cells. (a) Inhibition diagram of the effect of lidocaine on the Nav_1.5_ current in CHO cells. (b) Inhibition diagram of the effect of different DFD concentrations on Nav_1.5_ current in CHO cells. (c) Quantitative analysis of the inhibitory rate of DFD on Nav_1.5_ current in CHO cells (Tukey's multiple comparison test). (d) Dose-effect curve of the effect of a concentrated solution on Nav_1.5_ current in CHO cells. ^*∗*^*P* < 0.05,^*∗∗*^*P* < 0.01,^*∗∗∗∗*^*P* < 0.0001.

Different DFD concentrations, ranging from 0.8 to 24.0 mg/mL, inhibited the Nav_1.5_ current of CHO cells to varying degrees (Figure 3). The inhibition rate of 0.1 mmol/L lidocaine for Nav_1.5_ was 39.14 ± 3.96% (Figure 4(a)). The higher the concentration, the more obvious the inhibitory effect (Figure 4(b)). DFD suppressed Nav_1.5_dose-dependently with an IC_50_ of 24.0 ± 2.4 mg/mL (Figures 4(c) and (d)).

## 4. Discussion

Over time, the prevention and treatment of arrhythmias are evolving, and early medical therapy and ablation guided by electrophysiology have been replaced by implantable cardioverter defibrillators [11]. However, their relatively high cost limits their use and promotion, and thus, all patients are not benefitted. As the treatment of arrhythmias becomes increasingly diverse, the use of Chinese herbal medicines has spread from home to abroad and they are being widely and frequently used [12, 13]. Wenxin Keli and Shensong Yangxin capsules are commonly used [14], and their antiarrhythmic mechanisms are being discovered [15–17]. DFD, as a noninvasive therapy, is widely used in hospitals and has a good curative effect [6]. Herein, we illustrated that DFD protected against the barium chloride- and aconitine-induced VA and oxygen free radicals. Moreover, we showed that the mitigation effects of DFD were mediated by inhibiting Nav_1.5_ current.

In the ECG study, we found that DFD prolonged the occurrence and shortened the duration of VA in rats with VA induced by barium chloride and aconitine. Therefore, we deduced that DFD may be related to inward rectifier potassium channel, and it can prevent and postpone arrhythmia by promoting K^+^ outflow and reducing intracellular positive potential and automaticity. It may also be associated with a fast Na^+^, and it may prevent arrhythmias by inhibiting Na^+^ influx and reducing the fast response of cellular autonomy. Studies have shown that oxidative stress is one of the causes of arrhythmias [18]. Our results showed that DFD may reduce the content of MDA and increase the activity of SOD in rats with VA, by having the effect of antioxidant free radicals to protect the cardiomyocytes.

Cardiomyocytes are almost entirely dependent on aerobic metabolism. Na^+^-K^+^-ATPase is an omnipresent biofilm enzyme system on the cell membrane of the body [19]. Na^+^-K^+^-ATPase can regulate the concentration of sodium/potassium ion in cells, thus maintaining the resting potential of cells. Maintaining the activity of Na^+^-K^+^-ATPase helps to maintain the normal physiological function of cells. While further exploring the mechanism of DFD in arrhythmia, we found that DFD can protect Na^+^-K^+^-ATPase, an important indicator to monitor the occurrence of VAs [20]. Cx43 is the most abundant protein in the formation of gap junction pores in cardiomyocytes, which plays a vital role in the propagation of normal action potentials [21]. The decrease in Cx43 can reduce the coupling between myocytes, and it can also lead to an uneven action potential duration, thus inducing VAs [22]. Both the quantity and distribution of Cx43 are likely to cause abnormal electrical conduction velocity and heterogeneity between ventricular myocytes, which is particularly likely to result in the occurrence of VAs. Moreover, lowering the intracellular redox potential increases the opening of Cx43 [23]. Our results showed that one of the mechanisms of DFD in preventing and postponing VAs may be achieved by increasing the expression of Cx43 protein in left ventricular myocytes. To further explore the electrophysiological mechanism of DFD in arrhythmia, we conducted a whole-cell patch-clamp experiment and found that DFD inhibited the Nav_1.5_dose-dependently with an IC_50_ of 24.0 ± 2.4 mg/mL. There is strong evidence that DFD is a clinically effective antiarrhythmic medicine for treating VPC [6]. Thus, we studied the effect of DFD in animal arrhythmia models, which provided valuable laboratory research data. Additionally, absence of Torsade de Pointes or other new types of arrhythmias and the dose-dependent effect indicates the safety property of DFD.

Ion channels are critical for all aspects of cardiac function, including rhythmicity and contractility [24]. Indeed, most antiarrhythmic drugs modulate ion channel conductivity [25]. The structures, voltage sensor movements, and regulation of ion channels have been reported [26–28], which may help us to elucidate the disease mechanisms and potential therapeutic targets for future drug development. In this study, we determined the effect of DFD on Nav_1.5_ current, which generates cardiac action potentials and initiates the heartbeat [26], but we did not investigate the other ion channels, although other ion channels also play a role in arrhythmias. In view of the multicomponent nature of DFD, it may also affect other ion channels, like Xinsuning [7]. In our future report, we aim to show the multi-ion channel mechanism of DFD. In addition, with the development of mass spectrometry, multiple constituents of the components of DFD have been analyzed and characterized in recent years [29–34]. Modern pharmacological studies have shown that the components of DFD have anti-inflammatory [35–39], antioxidative stress [40–42], and myocardial protection [42–45] effects. Inflammation and oxidative stress are closely related to arrhythmia [46–48]. Moreover, *Radix Polygalae* plays an antiarrhythmic role by inhibiting the triggered activities in cardiomyocytes [45]. All of these findings suggest the antiarrhythmic mechanisms of DFD. In the future research, we will use ultraperformance liquid chromatography-quadrupole time-of-flight mass spectrometry (UPLC-QTOF-MS) to further clarify and identify the multiple components of DFD.

The results presented in this study improved our understanding of the clinical antiarrhythmic action of DFD, which is harmonized to obtain the therapeutic efficacy without causing any side effects. It is known that arrhythmia is caused by other underlying heart diseases and is a multifactorial disease requiring multicomponent medication. In this study, we found that DFD, a multicomponent medicine, inhibits arrhythmias and displays clear cellular electrophysiological mechanisms that support its clinical efficacy, and the results are similar to the properties shown by antiarrhythmic chemical drugs, although the data present the overall effect of a multicomponent medicine.

## 5. Conclusions

DFD conclusively showed the mechanism of the class I antiarrhythmic properties and the safety profile. The discovery that DFD mitigates oxidative stress, alleviates the activation of Na^+^-K^+^-ATPase and Cx43, and blocks Nav_1.5_current shows the cellular electrophysiological mechanisms that support the clinical therapeutic effect of DFD.

## Data Availability

The raw data used to support the findings of this study were supplied by Hui-Ling Liao under license and so cannot be made freely available. Requests for access to these data should be made to Hui-Ling Liao, liaohl@swmu.edu.cn.
